# Trauma-related intrusive memories and anterior hippocampus structural covariance: an ecological momentary assessment study in posttraumatic stress disorder

**DOI:** 10.1038/s41398-024-02795-1

**Published:** 2024-02-02

**Authors:** Quentin Devignes, Boyu Ren, Kevin J. Clancy, Kristin Howell, Yara Pollmann, Lucia Martinez-Sanchez, Courtney Beard, Poornima Kumar, Isabelle M. Rosso

**Affiliations:** 1 Center for Depression, Anxiety and Stress Disorders, McLean Hospital, Belmont, MA USA; 2 Department of Psychiatry, Harvard Medical School, Boston, MA USA; 3 Psychiatric Biostatistics Laboratory, McLean Hospital, Belmont, MA USA; 4 Division of Depression and Anxiety Disorders, McLean Hospital, Belmont, MA USA

**Keywords:** Hippocampus, Human behaviour

## Abstract

Trauma-related intrusive memories (TR-IMs) are hallmark symptoms of posttraumatic stress disorder (PTSD), but their neural correlates remain partly unknown. Given its role in autobiographical memory, the hippocampus may play a critical role in TR-IM neurophysiology. The anterior and posterior hippocampi are known to have partially distinct functions, including during retrieval of autobiographical memories. This study aimed to investigate the relationship between TR-IM frequency and the anterior and posterior hippocampi morphology in PTSD. Ninety-three trauma-exposed adults completed daily ecological momentary assessments for fourteen days to capture their TR-IM frequency. Participants then underwent anatomical magnetic resonance imaging to obtain measures of anterior and posterior hippocampal volumes. Partial least squares analysis was applied to identify a structural covariance network that differentiated the anterior and posterior hippocampi. Poisson regression models examined the relationship of TR-IM frequency with anterior and posterior hippocampal volumes and the resulting structural covariance network. Results revealed no significant relationship of TR-IM frequency with hippocampal volumes. However, TR-IM frequency was significantly negatively correlated with the expression of a structural covariance pattern specifically associated with the anterior hippocampus volume. This association remained significant after accounting for the severity of PTSD symptoms other than intrusion symptoms. The network included the bilateral inferior temporal gyri, superior frontal gyri, precuneus, and fusiform gyri. These novel findings indicate that higher TR-IM frequency in individuals with PTSD is associated with lower structural covariance between the anterior hippocampus and other brain regions involved in autobiographical memory, shedding light on the neural correlates underlying this core symptom of PTSD.

## Introduction

Recurrent and persistent trauma-related intrusive memories (TR-IMs) are core clinical symptoms of posttraumatic stress disorder (PTSD) [1, 2]. TR-IMs are defined as memories related to a traumatic event that spontaneously come to mind without volitional retrieval [3, 4]. Studies have shown that TR-IMs in the aftermath of a traumatic experience are strong predictors of the development and severity of other PTSD symptoms [5–8]. Moreover, network analyses of symptom associations in trauma-exposed individuals have demonstrated that intrusion symptoms, especially TR-IMs, are centrally connected to other symptoms such as avoidance and physical reactivity [9–11]. Network analyses have also shown that intrusion symptoms are associated with impairment in social and daily life functioning [7, 12]. In sum, TR-IMs are central symptoms of PTSD and may be critical treatment targets [13, 14].

The severity of intrusion symptoms is typically measured by their frequency and intensity (i.e., the distress they cause) [1, 15]. Most studies assessing TR-IM frequency have relied on retrospective reports [16–18]. However, this approach is limited by inherent biases in memory recall, including cognitive biases and the influence of mood and context at the time of recall [19]. Moreover, PTSD has been associated with disturbances in autobiographical memory, potentially further compromising the reliability of retrospective reports of TR-IMs [20, 21]. To overcome these limitations, ecological momentary assessments (EMAs) can be employed to assess TR-IM frequency [19, 22]. EMAs involve repeated assessments of a particular behavior or phenomenon at strategically predefined times and over a given period. EMAs capture real-time information from participants’ daily life [19, 22] and thereby produce data that are more ecologically valid than data obtained using retrospective assessments. Although positive correlations have been found between TR-IM frequency assessed using retrospective questionnaires and EMA in PTSD [23–25], one study reported that EMA identified about 50% more TR-IMs than retrospective measures [23]. Thus, EMA is a powerful method with more sensitivity and accuracy for detecting TR-IM frequency than retrospective reports. Combined with neuroimaging, EMA may lead to new insights on TR-IM neural correlates in PTSD.

The hippocampus (HPC) may play a key role in the neurophysiology of TR-IMs given its fundamental importance in autobiographical memory [26, 27] and well-validated significance in the neural circuitry of PTSD [28, 29]. Meta-analyses have consistently implicated smaller HPC volumes in PTSD patients compared to non-PTSD trauma-exposed and healthy individuals [30–33]. Regarding TR-IMs, some studies have found a negative association of HPC volume with intrusion symptom severity in PTSD patients [34–38], while others have not [39–41]. These discrepancies may arise from considering the HPC as a unitary structure. Indeed, a functional dissociation of the HPC into anterior (aHPC) and posterior (pHPC) subregions along its longitudinal axis (ventral and dorsal subregions in rodent models, respectively) was proposed as early as the first lesion studies of this structure more than half a century ago [42, 43]. Since then, evidence from preclinical and clinical studies has shown that the aHPC and pHPC have partially distinct structural and functional patterns of connections with other brain areas, consistent with different functions for these subregions [44–47]. In the context of autobiographical memory, the aHPC is proposed to play a predominant role in the initial searching and accessing of memories (the construction stage), while the pHPC may be primarily involved in elaborating episodic details (the elaboration stage) [27, 48–50]. Moreover, repeated retrieval of autobiographical memories engages the aHPC and pHPC differently, with decreased activation in the aHPC during repeated retrieval, but not in the pHPC [51, 52]. Furthermore, top-down inhibitory modulation of the right dorsolateral prefrontal cortex on aHPC activity during attempts to block intrusive memories has been reported [53]. These findings in healthy adults highlight distinct functions of the aHPC and pHPC in retrieving autobiographical memories, underscoring the relevance of subdividing the HPC along its longitudinal axis to understand the neurophysiology of TR-IMs.

Despite the importance of differentiating the aHPC and pHPC to characterize memory functions, there has been little research on how the morphology of these subregions relates to trauma-reexperiencing symptoms. A study conducted on a pediatric PTSD sample found a significant negative correlation between right aHPC volume and intrusion symptom severity, but not with overall PTSD severity or the severity of other PTSD symptoms, suggesting a specific link with intrusion symptoms [54]. It is worth noting that previous studies investigating aHPC and pHPC volumes in PTSD have primarily used a regional approach, focusing on specific brain regions selected a priori [54–56]. However, it has been shown that morphologic features, such as volume and thickness, tend to covary across communities of brain regions, a phenomenon known as “structural covariance” [57]. Structural covariance network (SCN) analysis aims to uncover the relationships between morphological features of different brain regions at the group level. Importantly, the biological relevance of SCNs has been supported by studies reporting significant associations with functional and structural connectivity [57, 58]. Moreover, SCNs typically include brain regions involved in similar behavioral or cognitive functions [57]. Although alterations in SCNs have been observed in PTSD [59–63], most studies have focused on cortical thickness [59, 62]. To our knowledge, only one study included the HPC and reported reduced covariance between ipsilateral HPC and amygdala volumes in PTSD patients compared to healthy controls [63]. However, this study did not distinguish between aHPC and pHPC. Furthermore, no study has examined the relationship between aHPC/pHPC SCN and the severity of intrusion symptoms, particularly TR-IM frequency.

To address this gap, this study investigated the morphological neural correlates of TR-IM frequency by using EMA with aHPC/pHPC volumes and SCN in a sample of trauma-exposed adults with varying severity of PTSD symptoms. Based on previous research associating autobiographical memory functions with the anterior-posterior axis of the HPC, the aHPC and pHPC were selected as the regions of interest for volumetric analyses and as seeds for the structural covariance approach. Given the predominant involvement of the aHPC in the construction and retrieval of autobiographical memories, we hypothesized that: (1) higher TR-IM frequency would be associated with smaller aHPC volume; (2) higher TR-IM frequency would be associated with lower structural covariance between the aHPC and brain regions involved in autobiographical memory. We also examined whether significant relationships between TR-IM and HPC morphology were specific to TR-IMs (intrusion symptoms), remaining significant after controlling for the severity of other PTSD symptoms.

## Materials and methods

### Participants

Trauma-exposed adults (*N* = 104) were recruited via advertisements in the local community and through McLean Hospital, including the Behavioral Health Partial Hospital Program and outpatient trauma programs. Study procedures were approved by the Mass General Brigham Human Research Committee, and all participants provided written and oral informed consent. The inclusion criteria were: (a) 18 to 65 years of age; (b) exposure to at least one DSM-5 Criterion A trauma for PTSD; (c) endorsement of at least two TR-IMs per week in the past month; (d) sufficient proficiency in English to complete study procedures; (e) access to a smartphone compatible with the MetricWire application (MetricWire Inc., Kitchener, Ontario, Canada). In addition, the exclusion criteria were: (a) left-handedness; (b) medical condition that could confound results (e.g., seizure disorder); (c) current psychotic disorder or manic mood episode; (d) history of moderate-to-severe traumatic brain injury, or head trauma with loss of consciousness >5 min; (e) past month moderate-to-severe alcohol or substance use disorder; (f) contraindications for magnetic resonance imaging (MRI); (g) positive pregnancy test for female participants on the day of MRI scanning; (h) report of experiencing intrusions only as thoughts, not as memories; (i) completion of less than 70% of the daily surveys (i.e., 49 surveys) during the EMA period. This cut-off value was based on precedent in the literature, particularly on our prior work using EMA for stress-related symptoms in clinical samples of similar severity [64]. Finally, to assess the exclusion criterion about experiencing intrusions only as thoughts, intrusive memories were defined at the beginning and then multiple times throughout the course of the study based on precedence from prior research [65], as follows: “*Sometimes people experience intrusive memories of their traumatic experience. These memories may pop into their mind at times when they do not want them to. Your most intense or vivid unwanted memories might make you feel like you are watching a movie screen or seeing a snapshot, hearing words or other sounds, or experiencing bodily sensations*.” This definition [65] was provided on the online pre-screen, the phone screen, and in the written instructions of self-report measures that assessed intrusive memory characteristics. In addition, participants completed the original version of the Intrusion Questionnaire [65] at visits 1 and 2. In this questionnaire, they additionally were asked: “*How do you experience this memory? Does it involve (check all that apply): (a) feelings or emotions; (b) sensory experiences; (c) thoughts; (d) bodily sensations*.” If a participant only checked “thoughts”, they were excluded from the study.

The study included two visits separated by a two-week EMA period. During the first visit, participants provided informed consent, completed a demographic information form, and filled out self-report questionnaires, including the PTSD Checklist for DSM-5 (PCL-5) [66]. The two-week EMA period, described below, started the day following the first visit. The second visit took place within two weeks after the EMA period’s completion for participants who had completed at least 70% of their EMA surveys. This visit involved clinical interviews, self-report measures, and MRI scanning. Eleven (11) participants were excluded from the analyses because they were either missing the MRI scan (*N* = 5), missing the T2-weighted turbo-spin echo MRI sequence needed for HPC segmentation (*N* = 5), or had structural brain abnormalities that would confound results (*N* = 1). Table 1 shows the demographic and clinical characteristics of our final sample of 93 participants. Importantly, when comparing our final sample (*N* = 93) to participants excluded from the study (*N* = 19) due to completing less than 70% of daily surveys, no significant differences were found in terms of age (*p* = 0.589), overall PTSD symptom severity measured by the PCL-5 total score at baseline (*p* = 0.799), and number of TR-IMs per day (*p* = 0.769) using unpaired two-samples Wilcoxon rank sum test. There also was no significant group difference in sex distribution (*p* = 0.539) using Fisher’s exact test.

**Table 1:** Demographic and clinical characteristics of the sample (*N* = 93).

Characteristic	Mean (SD) or *N* (%)
Age	32.7 (11.18)
Sex assigned at birth (female/male)	75 (80.65%)/18 (19.35%)
Race/ethnicity	
Asian	4 (4.30%)
Bi-/multiracial	17 (18.28%)
Black	5 (5.38%)
White	64 (68.82%)
Other	3 (3.23%)
Full PTSD diagnosis/subthreshold PTSD	73 (78.49%)/20 (21.51%)
MINI diagnosis	
Major Depressive Disorder	41 (44.09%)
Panic Disorder	9 (9.68%)
Agoraphobia	13 (13.98%)
Social Anxiety Disorder	13 (13.98%)
Generalized Anxiety Disorder	31 (33.33%)
Medication	
Antipsychotics	5 (5.38%)
Mood stabilizers	9 (9.68%)
Antidepressants	40 (43.01%)
Sedative-hypnotics	13 (13.98%)
Psychostimulants	16 (17.20%)
CAPS-5 composite score^a^	24.37 (9.27)
TR-IM frequency	21.35 (24.25)
Total number of surveys completed (out of 70)	58.4 (6.70)
Total number of TR-IM surveys completed (out of 42)^b^	35.26 (4.02)

Means (standard deviations) are presented for continuous variables and number of participants (percentage) for categorical variables.

*CAPS-5* Clinician-Administered PTSD Scale for DSM-5, *MINI* Mini International Neuropsychiatric Interview, *PTSD* posttraumatic stress disorder, *SD* standard deviation, *TR-IMs* trauma-related intrusive memories.

^a^Sum of scores associated with clusters C, D, and E.

^b^Only surveys assessing TR-IMs were included.

### Clinical interviews

*Clinician-Administered PTSD Scale for DSM-5 (CAPS-5)* – Doctoral-level clinicians administered the CAPS-5 [15], the gold-standard interview to determine PTSD diagnostic status and symptom severity. We derived a CAPS-5 composite score representing the severity of all non-intrusion symptoms by summing the scores for symptom clusters C (avoidance), D (negative thoughts and feelings), and E (arousal/reactivity).

*Mini International Neuropsychiatric Interview (M.I.N.I.)* – Doctoral-level clinicians administered the M.I.N.I. [67] version 7.0 in order to assess DSM-5 disorders including those relevant to the exclusion criteria.

### Ecological momentary assessments (EMA)

EMA was conducted using the MetricWire smartphone application (MetricWire Inc., Kitchener, Ontario, Canada). At the conclusion of the first study visit, participants received an orientation to the app and were assisted in downloading it onto their phone. Participants then were asked to complete five daily surveys over the following fourteen consecutive days, for a maximum of seventy surveys. The first daily survey was sent to participants at a random time within a three-hour window, commencing one hour before their usual wake time. The survey queried the occurrence of trauma-related nightmares the night prior. The three subsequent surveys were also distributed randomly within each consecutive three-hour block throughout the day and queried the occurrence of TR-IMs (“*Since the last time you completed a survey, did you have any unwanted memories of your trauma? (If “Yes”: How many?”)*. Finally, the fifth daily survey evaluated the severity of PTSD symptoms experienced over the past twenty-four hours using an adapted version of the PCL-5 [66]. Each survey was available for completion for 1.5 h. Participants’ compliance was monitored daily by a research assistant logging into the MetricWire website. In instances where a participant had not completed any surveys for two consecutive days, the research assistant contacted them via phone, aiming to ensure there was no technical issue preventing survey completion and/or to provide reminders for continued engagement. For this study, only the data from the three surveys assessing TR-IMs were analyzed, for a maximum of 42 TR-IM surveys over the two-week EMA period. We calculated TR-IM frequency for each participant as the total number of TR-IMs reported across all surveys during the two-week EMA period.

### Imaging acquisition

Participants were scanned at the McLean Hospital Imaging Center on a 3 T Siemens MAGNETOM Prisma scanner (Siemens Healthineers, Erlangen, Germany) with a 64-channel head coil. We used the Human Connectome Project (https://www.humanconnectome.org/) imaging protocol, including (a) a high-resolution MPRAGE T1-weighted sequence and (b) an ultra-high-resolution T2-weighted turbo-spin-echo sequence with slices oriented perpendicular to the long axis of the HPC (from the anterior margin of the amygdala to the most posterior part of the hippocampal tail). The high in-plane resolution of the T2-weighted sequence allows the identification of hippocampal subfields and amygdalar nuclei [68, 69]. More details on image acquisition and preprocessing can be found in the Supplementary Materials.

### Study-specific probabilistic templates

Because most atlases are generated from data acquired in healthy volunteers and HPC volumes are altered in PTSD compared to healthy controls [30–33], we created study-specific aHPC and pHPC templates using the following steps: (a) preprocessing and segmentation of T1-weighted images using Freesurfer software version 7.2 (https://surfer.nmr.mgh.harvard.edu); (b) segmentation of hippocampal subfields with the hippocampal subfield segmentation module [70] in Freesurfer using both T1- and T2-weighted sequences; (c) quality check of the segmentations; (d) aggregation of the hippocampal subfields to create an anterior subregion (head) and a posterior subregion (body + tail) according to previously published aggregation scheme [71–73]; (e) registration of the T1-weighted image of each participant to the Montreal Neurological Institute 152 (MNI152) 1 mm template; (f) application of the registration parameters to the aHPC and pHPC masks; (g) merging of aHPC and pHPC masks across participants to create probabilistic aHPC and pHPC templates. More details can be found in the Supplementary Materials.

**Fig. 1:**
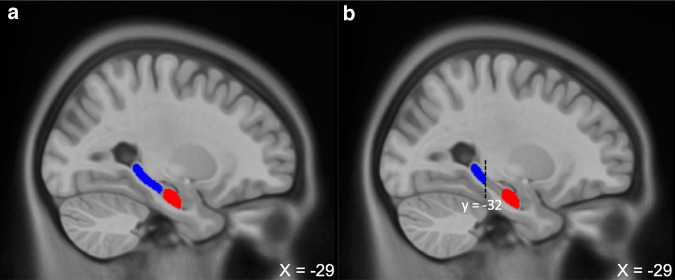
Study-specific templates of the anterior hippocampus (aHPC) and posterior hippocampus (pHPC). **a** Illustration shows left hemisphere aHPC (red) and pHPC (blue) templates in MNI space. **b** pHPC template was cut at *y* = −32 to remove the middle portion of the hippocampus. MNI Montreal Neurological Institute.

In the present study, we used templates with a probability of 0.75 (i.e., only voxels present at least in 75% of our sample were retained) to avoid including inconsistent voxels (Fig. 1a). Because the middle portion of the HPC has been associated with a mix of aHPC and pHPC functions, it was excluded from Freesurfer’s template, which includes it in the definition of pHPC. Instead, the pHPC Freesurfer template was cut according to a previously used criterion (*y* = −32) [45, 74]. The Freesurfer aHPC template did not need to be modified, as it already respected the previously used criterion (*y* = −21) [45, 74] (Fig. 1b).

### Volumetric and structural covariance network (SCN) analyses

T1-weighted images were preprocessed using the Computational Anatomy Toolbox version 12.8.2 (CAT12; https://neuro-jena.github.io/cat/). Subsequently, an average gray matter (GM) template was created, and the probabilistic templates for aHPC and pHPC were registered to this average GM template. For more details, see Supplementary Materials.

For each participant, the volumes of aHPC and pHPC were determined by multiplying the average GM density within the subregion by the number of voxels composing that subregion and then summing left and right volumes.

Regarding the SCN analysis, a seed-based partial least squares (PLS) analysis was performed using PLSgui version 6.15 (https://www.rotman-baycrest.on.ca/index.php?section=84) in Matlab version R2022b (MathWorks Inc., Natick, MA, USA) to construct the SCN [75]. Seed-based PLS is a data-driven multivariate approach that identifies voxels across the whole brain whose GM density correlates with the GM density of a seed region across participants. This approach is suitable for analyzing large-scale SCNs and has been used previously in multiple studies to examine structural network integrity [76, 77], including studies using aHPC and pHPC as seed regions [78, 79]. One advantage of seed-based PLS is that it considers all voxels simultaneously, thereby avoiding issues associated with multiple statistical comparisons.

To create the SCN, the average GM density was computed for each participant within the bilateral aHPC and pHPC using the individual normalized, modulated but unsmoothed GM images and the respective seed templates. A between-subject matrix representing the covariance between the average GM density of the seeds and all the other brain voxels was then computed. The number of voxels included in this matrix was constrained by a binary mask derived from the average GM template generated using the individual GM images.

This matrix was then decomposed into a latent variable (LV) that identified a pattern of structural covariance. Importantly, because the focus was on identifying a pattern that distinguished aHPC and pHPC, we performed a non-rotated PLS analysis. This approach allowed the specification of an a priori contrast to identify voxels that showed significant differences in salience (i.e., weight) between aHPC and pHPC. The significance of the LV was determined through non-parametric permutation tests (*n* = 1000) using resampling without replacement. The reliability of each voxel’s contribution to the LV was established by 1000 bootstraps with replacement, estimating the standard error of each voxel’s weight on the LV. A threshold of *p* < 0.05 was considered significant for the LV. A bootstrap ratio (BSR) (i.e., the ratio of each weight to its standard error) greater than 3.3 or less than −3.3 (corresponding to a *p*-value of 0.001) was considered reliable for each voxel. The automated anatomical labeling atlas version 3 was used to identify the brain regions included in the structural pattern [80].

Finally, a composite structural covariance score, referred to as the “brain score”, was calculated for each participant. This score represented the strength with which each participant expressed the pattern identified by the LV and was calculated as the dot product of the GM voxel density in each participant’s normalized, modulated and smoothed GM image with the corresponding voxel weight in the LV pattern.

### Statistical analyses

Statistical analyses were conducted using R software version 4.2.2 [81], with a two-tailed *p* < 0.05 significance threshold. Details of a priori power and sample size calculations are provided in Supplementary Materials.

The relationship of TR-IM frequency with CAPS-5 intrusive symptom severity (cluster B) was evaluated using Pearson correlation.

Two Poisson regression models were constructed to assess aHPC and pHPC volume as predictors of TR-IM frequency. In these models, age, sex, and total number of TR-IM surveys were entered as covariates, and the duration of the follow-up as an offset term. The small correlation between the total number of TR-IM surveys and the duration of the follow-up (*r* = −0.08; *p* = 0.476) justified the inclusion of both variables in our Poisson models.

For SCN analyses, we first characterized the pattern of covariance captured by the LV by calculating Pearson’s correlations between LV brain scores and volumes of the aHPC and pHPC. We then constructed a Poisson regression model to assess the association between TR-IM frequency (outcome) and LV brain scores (predictor), controlling for age, sex, and total number of TR-IM surveys as covariates. Total intracranial volume was not included as a covariate because the GM images were modulated to account for individual head size. To aid interpretation of results, all predictors and covariates were centered, such that a one-unit increase or decrease corresponded to change of one standard deviation in the respective predictor or covariate.

For Poisson models that identified significant HPC predictors of TR-IM frequency, we added the CAPS-5 composite score (that excluded intrusion symptoms) to our models as a covariate to determine whether associations remained significant after controlling for non-intrusion symptoms.

## Results

### Characterization of TR-IM frequency over the EMA period

On average, participants completed 35.26 (standard deviation (SD) = 4.02; range = [26; 42]) surveys assessing TR-IM occurrence and endorsed 21.35 (SD = 24.25; range = [0; 153]) TR-IMs over the 2-week EMA period (Table 1). TR-IM frequency was not significantly associated with intrusive symptom severity measured by the CAPS-5 Cluster B score (*r* = 0.17; *p* = 0.102).

### Relationship of TR-IM frequency with aHPC and pHPC volumes

In the first Poisson regression model (Nagelkerke *R*^2^ = 0.092), aHPC volume was not a significant predictor of TR-IM frequency (incidence rate ratio (IRR) = 0.98; *p* = 0.492; 95% confidence interval (CI) = [0.94; 1.03]), nor were the effects of age (IRR = 1.01; *p* = 0.610; 95% CI = [0.97; 1.06]) and sex (IRR = 0.94; *p* = 0.342; 95% CI = [0.84; 0.94]). However, the total number of TR-IM surveys completed was a significant covariate in the model (IRR = 0.94; *p* = 0.008; 95% CI = [0.90; 0.98]).

The second Poisson regression model (Nagelkerke *R*^2^ = 0.111) revealed that pHPC volume was not a significant predictor of TR-IM frequency (IRR = 1.04; *p* = 0.123; 95% CI = [0.99; 1.09]), and that neither age (IRR = 1.02; *p* = 0.417; 95% CI = [0.97; 1.06]) nor sex (IRR = 0.92; *p* = 0.159; 95% CI = [0.82; 1.03]) were significant covariates. In contrast, the total number of TR-IM surveys completed was a significant covariate (IRR = 0.94; *p* = 0.008; 95% CI = [0.90; 0.98]).

### Characterization of the pattern of structural covariance captured by the LV

**Fig. 2:**
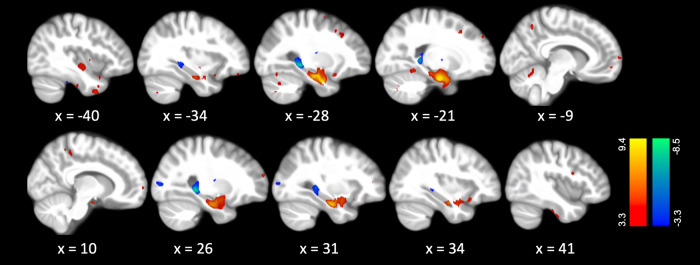
Structural covariance pattern identified by the seed-based partial least squares analysis. Color scales represent bootstrap ratios (proportional to z-scores). Brain regions that covaried positively with aHPC volume have positive bootstrap ratios (warm colors), while brain regions that covaried negatively with aHPC volume have negative bootstrap ratios (cold colors). aHPC anterior hippocampus.

PLS analysis identified a significant LV corresponding to the specified contrast (i.e., aHPC vs. pHPC volume) (*p* = 0.013). Pearson correlations showed that LV brain scores were significantly associated with aHPC volume (*r* = 0.60; *p* < 0.001) and not significantly associated with pHPC volume (r = −0.11; *p* = 0.288). Therefore, the LV brain scores represented a pattern of covariance with aHPC volume only. Brain regions showing positive covariance with aHPC included the left inferior and superior temporal gyri, bilateral superior frontal gyri, left middle frontal gyrus, bilateral fusiform gyri, bilateral precuneus, and bilateral cerebellar regions (Fig. 2 and Table 2). Fewer brain regions demonstrated negative covariance with aHPC volume, with the pHPC and right middle occipital gyrus being the most prominent regions (Fig. 2 and Table 2).

**Table 2:** Brain regions encompassed in the structural covariance pattern identified by the seed-based partial least squares analysis.

Label	Peak MNI coordinates			Number of voxels	Bootstrap ratio
	X	Y	Z		
Positive saliences					
Left parahippocampal gyrus	−28	−2	−30	7740	9.40
Right hippocampus (anterior)	31	−11	−24	6236	7.79
Left inferior temporal gyrus	−51	−15	−42	1141	5.32
Left inferior temporal gyrus	−43	8	−43	775	5.31
Left insula	−25	16	−17	230	4.73
Left superior temporal gyrus	−44	−10	−7	881	4.56
Left precuneus	−11	−59	52	178	4.46
Right precuneus	6	−40	52	525	4.44
Left superior frontal gyrus, dorsolateral	−13	67	9	245	4.43
Left fusiform gyrus	−22	−49	−12	197	4.34
Right superior frontal gyrus, dorsolateral	28	63	20	343	4.33
Left middle frontal gyrus	−31	28	40	552	4.28
Left fusiform gyrus	−37	−14	−34	258	4.26
Left postcentral gyrus	−53	−9	40	182	4.09
Right fusiform gyrus	38	−16	−40	762	4.04
Left lobule IV, V of cerebellar hemisphere	−6	−60	−16	416	4.00
Right lobule IV, V of cerebellar hemisphere	20	−50	−16	359	3.92
Right lobule VI of cerebellar hemisphere	17	−70	−17	518	3.88
Left Crus I of cerebellar hemisphere	−17	−90	−23	102	3.77
Right inferior frontal gyrus, opercular part	42	8	23	102	3.70
Left Crus I of cerebellar hemisphere	−17	−71	−35	260	3.65
Negative saliences					
Left hippocampus (posterior)	−26	−37	−3	1886	−8.46
Right hippocampus (posterior)	25	−37	−1	1753	−7.65
Right middle occipital gyrus	27	−90	6	328	−4.76

Labels, coordinates, and bootstrap ratios are reported for the peak voxel of each cluster. Positive saliences (i.e., weights) represent voxels covarying positively with aHPC, and negative saliences voxels covarying negatively with aHPC. Only clusters with at least 100 voxels are reported.

*aHPC* anterior hippocampus, *MNI* Montreal Neurological Institute, *pHPC* posterior hippocampus.

### Relationship of TR-IM frequency with LV brain scores

Poisson regression (Nagelkerke *R*^2^ = 0.179) revealed that TR-IM frequency was significantly associated with LV brain scores. Each unit decrease in LV brain scores was associated with an average increase of 8.61% in TR-IM frequency (IRR = 0.91; *p* = 0.002; 95% CI = [0.86; 0.97]). The total number of TR-IM surveys completed was a significant covariate in the model (IRR = 0.95; *p* = 0.018; 95% CI = [0.90; 0.99]), whereas the effects of age (IRR = 0.96; *p* = 0.194; 95% CI = [0.91; 1.02]) and sex (IRR = 0.98; *p* = 0.683; 95% CI = [0.87; 1.09]) were not statistically significant.

The association of LV brain scores with TR-IM frequency remained significant (IRR = 0.89; *p* < 0.001; 95% CI = [0.84; 0.94]) after adding the CAPS-5 symptom composite score as a covariate in the model (Nagelkerke *R*^2^ = 0.873).

## Discussion

Trauma-related intrusive memories are central symptoms and critical treatment targets in PTSD, but their neural correlates are not fully understood. The results of this study provide new insights into the relationship between the frequency of these memories and the morphology of aHPC and pHPC. To comprehensively characterize this relationship, we performed both a regional-based analysis using aHPC and pHPC volumes, and a network-based analysis by identifying the aHPC/pHPC SCN. We found no significant association of TR-IM frequency with aHPC and pHPC volumes. However, we identified a structural covariance pattern that was uniquely associated with aHPC volume, and the expression of this pattern was negatively correlated with TR-IM frequency. This result demonstrates that a higher frequency of TR-IMs is associated with lower structural synchronization between aHPC and brain regions involved in autobiographical memory. Importantly, this association remained significant after accounting for the severity of PTSD symptoms other than intrusion symptoms. This robust result underscores the specificity of our findings to intrusive reexperiencing, shedding light on the unique neural correlates underlying these core symptoms of PTSD. Our findings also highlight the relevance of a network-based approach over a regional approach in the study of TR-IM morphological neural correlates.

### PLS analysis

The PLS analysis yielded a significant structural covariance pattern uniquely associated with the volume of the aHPC. This finding aligns with a previous study that used the same statistical approach and identified a structural covariance pattern distinguishing aHPC and pHPC in healthy volunteers [82]. These results suggest that the aHPC displays greater unique whole-brain covariance than the pHPC.

The observed structural covariance pattern encompassed multiple temporal and frontal lobe gyri, which was expected given the known interaction between the aHPC and these regions during the initial construction phase of autobiographical memory retrieval [48]. Furthermore, a significant cluster was found in the right dorsolateral prefrontal cortex, displaying significant covariance with the aHPC. This prefrontal region has been implicated in top-down inhibition of aHPC activity during attempts to suppress intrusive memories in healthy volunteers [53]. Hence, the structural pattern identified in the present study closely mirrored the functional networks associated with autobiographical memory, aligning with previous research showing that inter-regional structural covariance partly contributes to functional connectivity [57].

The SCN identified in our study also involved other brain regions, particularly multiple cerebellar regions. Interestingly, a previous study of healthy individuals examining the pattern of structural covariance with aHPC volume did not identify any significant cluster in the cerebellum [82]. Nevertheless, the presence of significant structural covariance between the HPC and the cerebellum is not surprising. A growing body of evidence highlights the cerebellum’s role in emotional and cognitive processing [83, 84], including in retrieving autobiographical memories [85]. In addition, preclinical and clinical studies have demonstrated structural and functional connectivity between the cerebellum and HPC [86, 87]. Our findings extend these observations by revealing significant positive covariance between multiple cerebellar regions and the aHPC in a PTSD sample. Further investigations in healthy volunteers will be necessary to determine whether this relationship is specific to PTSD or trauma-exposed groups, or applies more broadly.

In line with our hypothesis, the main finding of the present study revealed a significant negative association between TR-IM frequency and LV brain scores. This negative association remained significant after controlling for the severity of PTSD symptoms other than intrusion symptoms, namely avoidance, negative alterations of cognitions and mood, and arousal/reactivity. This indicates that the observed relationship is specific to trauma-reexperiencing. This finding suggests that individuals who exhibit lower expression of the identified structural covariance pattern have more frequent TR-IMs, indicating that lower structural synchronization between brain regions involved in autobiographical memory is associated with a higher occurrence of intrusive memories related to a traumatic event. Interestingly, a previous study in healthy volunteers reported a significant positive correlation between the expression of aHPC SCN and associative memory performance [82]. Notably, associative learning and autobiographical memory involve overlapping brain regions, including the HPC, which is crucial in encoding contextual information associated with the traumatic experience [88, 89]. Collectively, these findings suggest that decreased structural synchronization between brain regions involved in associative learning and memory may be linked to dysfunction of these cognitive processes. However, it is important to note that our study did not include associative memory tasks, warranting further investigations to explore the relationship between reduced aHPC structural covariance and memory performance. Moreover, our cross-sectional study design cannot determine the directionality of the relationship between structural brain covariance and TR-IMs; it is possible that lower covariance contributes to more frequent TR-IMs and/or that higher TR-IM frequency has an impact on brain covariance patterns. Further studies are needed to elucidate potential causal relationships between TR-IM frequency and decreased structural covariance of the aHPC with other brain regions involved in autobiographical memory.

### Volumetric analysis

Contrary to our hypothesis, we did not uncover a significant association between TR-IM frequency and volumes of the aHPC or pHPC. Although meta-analyses have established that smaller HPC volume is a robust finding in PTSD [31–33], the relationship between HPC volume and intrusion symptoms remains unclear. Some studies have reported a significant negative correlation between HPC volume and intrusion symptom severity in PTSD adults [34–38], while others have not [39–41]. These inconsistencies may be partly explained by methodological differences across studies, including with the assessment of intrusion symptom severity and the neuroimaging techniques to segment the HPC.

Indeed, previous studies used different tools to estimate intrusion symptom severity. These have included clinician-administered interviews such as the CAPS-5 [15] and self-report questionnaires such as the PCL-5 [66]. Importantly, most of these previous studies estimated the overall severity of intrusion symptoms rather than specifically examining TR-IM frequency [34–38]. To our knowledge, only one study has directly investigated the relationship between TR-IM frequency and HPC volume [90]. Using a neurocomputational model, this study reported that the perceived danger of a traumatic event was positively associated with the likelihood of experiencing intrusive memories and that higher TR-IM frequency was linked to smaller HPC volume [90]. However, it is important to note that this study relied on simulated data rather than clinical data and did not control for potentially confounding demographic and clinical variables. Furthermore, most of the questionnaires used in previous studies include other intrusive phenomena, such as nightmares, within the reexperiencing/intrusion symptom category. These questionnaires typically asked participants to rate the level of distress or bother caused by intrusion symptoms using summary Likert scales, without directly estimating TR-IM frequency. Consequently, the specific relationship of HPC volume with TR-IM frequency, distinct from the overall severity of intrusion symptoms, has not yet been fully characterized in people with PTSD.

Regarding the segmentation techniques used for HPC volume analysis, it is worth noting that previous studies reporting no significant associations between intrusion symptom severity and HPC volume primarily employed the automatic segmentation provided by Freesurfer software [39–41]. Conversely, studies that observed significant associations mainly used manual segmentations [35–38]. In this study, we used the Freesurfer module for HPC subfields and amygdalar nuclei segmentation [70], which has been shown to provide reliable anatomical segmentations overall, except for the smallest sub-structures like the hippocampal fissure, which was not included in our aggregation scheme [91]. In addition, we used a multispectral approach known to optimize HPC segmentation, by combining a high-resolution T1-weighted sequence with an ultra-high-resolution T2-weighted sequence [92]. Therefore, the absence of significant associations between TR-IM frequency and HPC volumes in the present study aligns with previous investigations employing a similar segmentation technique [39–41].

Finally, it is important to highlight that prior studies of HPC volume in PTSD have rarely distinguished between aHPC and pHPC, leading to inconsistent findings [54–56, 93]. To our knowledge, only one previous study has investigated the relationship of intrusion symptom severity with aHPC and pHPC volumes [54]. This study reported a significant negative association with aHPC volume in a pediatric PTSD sample, but it considered an overall score of intrusion symptom severity and did not directly estimate TR-IM frequency [54]. Thus, our study contributes novel findings by demonstrating that TR-IM frequency is not significantly associated with aHPC and pHPC volumes in a sample of adults with PTSD. Together, the findings of the present study suggest that a higher TR-IM frequency is not related to the morphology of a singular brain structure but rather to the structural synchronization within a widespread brain network centered on the anterior hippocampus. In other words, a higher TR-IM frequency was not significantly associated with smaller or larger hippocampal volumes but with a lower synchronization of volumes between the aHPC and other brain regions involved in autobiographical memory: the volume of these brain regions covaried significantly less in participants experiencing more TR-IMs.

### Strengths and limitations

Our study possesses several strengths that increase confidence in the findings. First, we used EMA to estimate the frequency of TR-IMs. This approach stands out from previous studies that predominantly relied on retrospective reports, which are susceptive to various biases and inaccuracies [19]. Second, we created study-specific templates using ultra-high-resolution MRI images to precisely delimit the aHPC and pHPC. In contrast, previous studies of HPC SCN often relied on atlases generated from data of healthy participants [78, 79]. Finally, we applied PLS analysis as a powerful statistical method to identify large-scale covariance patterns without the issue associated with multiple comparisons.

While our study offers novel insights, it is important to acknowledge its limitations. First, the absence of healthy participants could raise the question of the generalizability of the structural covariance pattern identified in our study to non-traumatized individuals. However, it is noteworthy that the structural covariance pattern identified in our study closely resembles the one found in a study of healthy individuals using the same statistical approach [82]. Additionally, considering that over 70% of the general population has experienced at least one traumatic event [94], it is likely that some healthy participants included in this previous study were not trauma-naïve, a variable that was not assessed [82]. Second, further investigations are needed to determine whether our findings are specific to PTSD, or whether they extend to other psychiatric conditions with a high incidence of trauma exposure and intrusive memories, such as major depressive disorder. Moreover, our study included participants with subthreshold PTSD; however, the sample size of this subgroup was insufficient for conducting meaningful between-group comparisons. Given the prevalence of subthreshold PTSD among trauma-exposed individuals – approximately 14.7% – and its significant impact on occupational, psychosocial, and daily life impairment [95], future investigations should examine the replicability of our findings in subclinical trauma-exposed individuals who experience TR-IMs without meeting the formal criteria for PTSD diagnosis. Furthermore, as is inherent to studies of psychiatric samples skewed toward higher clinical severity and individuals meeting DSM-5 diagnostic criteria, our sample had a truncated range of symptom severity above a certain threshold, which reduces the likelihood of detecting associations with neurobehavioral correlates and further underscores the importance of including subthreshold participants. Understanding the generalizability of these findings across different psychiatric conditions and PTSD symptom severity could provide valuable insights into the underlying mechanisms of trauma-related psychopathology. Third, although our study sheds light on the neural correlates underlying TR-IM frequency, it does not provide information regarding the spatial progression of decreasing structural covariance between the brain regions encompassed in the identified pattern as TR-IM frequency increases. Recently, a study utilizing causal structural covariance network analysis reported that changes in the structural integrity of the HPC may potentially lead to structural alterations in frontal regions, and, subsequently, in temporal and occipital regions, highlighting the central role of HPC in structural covariance alterations in PTSD [96]. Speculatively, the spatial progression of the identified SCN in our study could follow a similar pattern, with the aHPC playing a central role. However, this previous study used PTSD symptoms severity, not TR-IM frequency, to create pseudo-time series [96]. Therefore, further investigation is needed to determine the spatial course of the aHPC SCN and its association with TR-IM frequency. Finally, although all aspects of the study design were defined a priori in grant and ethics applications, the hypotheses of the present study were not pre-registered publicly.

## Conclusions

Our study demonstrates for the first time that a higher frequency of TR-IMs is associated with lower structural synchronization within an anterior hippocampus-cortical network involved in autobiographical memory in a sample of adults with PTSD. This novel finding highlights the potential of structural covariance network analysis, especially focused on the anterior hippocampus, to identify biomarkers associated with recurrent trauma-related intrusive memories. To extend this line of inquiry, future investigations should explore whether this neural correlate represents a premorbid risk factor for greater TR-IM frequency, results from trauma exposure, or a combination of both.

### Supplementary information

Supplementary Materials

## Data Availability

Data are available through the NIMH National Data Archive (NDA; https://nda.nih.gov/edit_collection.html?id=3224) and are available upon reasonable request to the senior author, IMR.

## Code availability

Shareable analysis code is available on the open science framework data repository (https://osf.io/kavpc/?view_only=7f1af53a5b3647b18a45d023c91f02d5).
