# Supplementary material for: Trauma-related intrusive memories and anterior hippocampus structural covariance: an ecological momentary assessment study in posttraumatic stress disorder
Source: Transl Psychiatry. 2024 Feb 2;14:74. doi: 10.1038/s41398-024-02795-1 (PMC10837434; doi:10.1038/s41398-024-02795-1)
Supplement: Supplementary file 1 — Supplementary Materials [file 41398_2024_2795_MOESM1_ESM.docx]

**Supplementary materials**

**MATERIALS AND METHODS**

Imaging acquisition

Structural sequences were initially acquired using the Human Connectome Project (HCP; <https://www.humanconnectome.org/>) Young Adult protocol (n = 7 subjects). Early in the study, the imaging protocol was transitioned to the HCP Lifespan protocol (n = 86). These two protocols were developed to be compatible with each other [1]. The imaging protocol included: (a) an anatomical high-resolution MPRAGE T1-weighted sequence (parameters for the HCP Young Adult protocol: voxel size = 0.7 mm isotropic; TR = 2,400 ms; TE = 2.14 ms; FOV = 224 x 210 mm; matrix size = 208 x 300 x 320 slices; flip angle = 8°; parameters for the HCP Lifespan protocol: voxel size = 0.8 mm isotropic; TR = 2,500 ms; TEs = 1.81/3.6/5.39/7.27 ms; FOV = 256 x 240 mm; matrix size = 208 x 300 x 320 slices; flip angle = 8°) and (b) a high-resolution T2-weighted turbo-spin-echo sequence with slices oriented perpendicular to the long axis of the HPC (from the anterior margin of the amygdala to the most posterior part of the hippocampal tail) (parameters for the HCP Young Adult protocol: voxel size = 0.7 x 0.7 x 2 mm^3^; TR = 4,440 ms; TE = 84 ms; FOV = 230 x 187; matrix size = 260 x 320 x 36 slices; flip angle = 90°; parameters for the HCP Lifespan protocol: voxel size = 0.4 x 0.4 x 2.0 mm^3^; TR = 4,800 ms; TE = 106 ms; FOV = 150 x 150; matrix size = 384 x 384 x 30 slices; flip angle = 135°). Each image was visually inspected to ensure the absence of artifacts and anatomical abnormalities before inclusion in the analyses, resulting in no exclusion.

Study-specific probabilistic templates

Automated pre-processing and segmentation of T1-weighted images were performed using the recon-all pipeline with default settings from the Freesurfer software version 7.2 (<https://surfer.nmr.mgh.harvard.edu>). This pipeline includes the following steps [2,3]: motion correction, removal of non-brain tissue, Talairach transformation, intensity normalization, segmentation of subcortical gray and white matter, smoothing, tessellation of gray matter/white matter boundary, topology correction, surface deformation, cortical parcellation. More details are available at <https://surfer.nmr.mgh.harvard.edu/fswiki/recon-all>.

Automated segmentation of hippocampal subfields was performed using the hippocampal subfield segmentation module [4] in Freesurfer version 7.2. This module uses an ultra-high-resolution probabilistic atlas generated from *ex vivo* MRI data to segment the HPC into nineteen subfields. To obtain a reliable segmentation, we performed a multispectral segmentation using both T1- and T2-weighted sequences described above. This combination took advantage of the high resolution of the T2-weighted images, while the T1-weighted images provided complementary information in case of missing hippocampal voxels in the T2 scan due to its limited field of view. A comprehensive quality check of the segmentation was performed according to an adapted version of the pipeline recommended by the Enhancing Neuro Imaging Genetics through Meta-Analysis (ENIGMA) consortium [5]. Briefly, this pipeline includes: (a) a quality check of the cortical and subcortical segmentation (see <https://enigma.ini.usc.edu/protocols/imaging-protocols/> for details), (b) an identification of outliers based on the hippocampal subfield volumes (for each subfield, outliers were defined as having a volume inferior or superior to the average volume ± 2.68 standard deviations), (c) identification of outliers based on the ranking of certain subfield volumes (i.e., CA1 at rank #1; hippocampal tail below rank #3; subiculum at rank #4), and (d) a visual check of the hippocampal segmentation of each subject, with particular attention to the subjects with outliers. However, because of potential hippocampal atrophy that may impact the subfields differently in PTSD [6], we did not include the outlier identification based on ranking in our quality check pipeline. No participant was excluded based on the quality check.

Freesurfer’s segmentation module divides the hippocampus into three main subregions: head, body, and tail. Most of the hippocampal subfields have both a head and a body subdivision (e.g., molecular layer-head, molecular layer-body). To examine volumes along the longitudinal axis of the HPC, we aggregated the hippocampal subfields to create an anterior subregion (head) and a posterior subregion (body + tail). A similar aggregation scheme has been used in multiple other studies [7–9]. The aHPC volume was defined as the sum of the volumes of the head subdivision of the following subfields: Cornu Ammonis 1 (CA1), CA2/CA3, CA4, molecular layer, granule cell and molecular layer of the dentate gyrus (GC-ML-DG), subiculum and presubiculum. The pHPC volume was defined as the sum of the volumes of the body subdivision of the same subfields plus the hippocampal tail and the fimbria. Therefore, aHPC and pHPC volumes were composed of distinct portions of the hippocampal subfields: the two subregions were exclusive.

We used a two-step process to register the T1-weighted image of each participant to the Montreal Neurological Institute 152 (MNI152) 1mm template: (a) an affine registration with the correlation ratio cost function and trilinear interpolation using FMRIB’s Linear Image Registration Tool (FLIRT) version 6.0 [10], and (b) a diffeomorphic registration using the symmetric normalization (SyN) algorithm and B-Spline interpolation in Advanced Normalization Tools (ANTs) version 2.4.3 [11]. The parameters from the two registrations were then applied to the aHPC and pHPC masks using FLIRT trilinear interpolation followed by MultiLabel interpolation in ANTs. Finally, aHPC and pHPC masks were merged across participants to create probabilistic aHPC and pHPC templates.

Structural covariance network (SCN) analysis

T1-weighted images were preprocessed using the Computational Anatomy Toolbox version 12.8.2 (CAT12; <https://neuro-jena.github.io/cat/>) implemented in MATLAB version R2023a (MathWorks Inc., Natick, MA, USA). The following steps were applied to each participant’s image: (a) spatial normalization to MNI152 space using geodesic shooting registration [12]; (b) tissue classification into grey matter (GM), white matter, and cerebrospinal fluid; (c) bias correction of intensity non-uniformities; (d) non-linear modulation to compute the absolute amount of brain tissue (i.e., GM density), corrected for individual head size; (e) smoothing with a 6mm full-width at half-maximum Gaussian kernel, with the resulting voxel size of 1mm^3^.

The quality assessment of the raw T1-weighted images was conducted using the weighted average image quality rating implemented in CAT12. All images received a quality rating of C+ (i.e., satisfactory) or above, and therefore no participant was excluded. Subsequently, normalized, modulated but unsmoothed GM images were averaged across all participants using the Masking toolbox [13] in Statistical Parametric Mapping version 12 (SPM12; <https://www.fil.ion.ucl.ac.uk/spm/>) to create an average GM template. This whole-brain template was then thresholded at 0.15 to exclude non-GM areas. The probabilistic templates for aHPC and pHPC were registered to the average GM template using a trilinear interpolation in SPM12 and were thresholded at 0.9.

Power and sample size analysis

To estimate the sample size needed for 3 two-tailed Poisson regression models, we used WebPower R package (<https://cran.r-project.org/web/packages/WebPower/WebPower.pdf>) with the following parameters:

- Significance level of α = 0.05/3 = 0.017
- Base rate of 1 (i.e., 1 TR-IM per day)
- Effect sizes (i.e., incidence rate ratio (IRR)) varying from 1.68 (small) to 3.47 (medium) to 6.71 (large) [14]
- Power of 80%

The plot below represents the minimum sample size as a function of the effect size with the abovementioned parameters. The dashed red line represents the sample size of the present study (N = 93). This plot illustrates that the sample size of the present study (N = 93) was sufficient to detect small to medium effect sizes with a power of 80%.

**
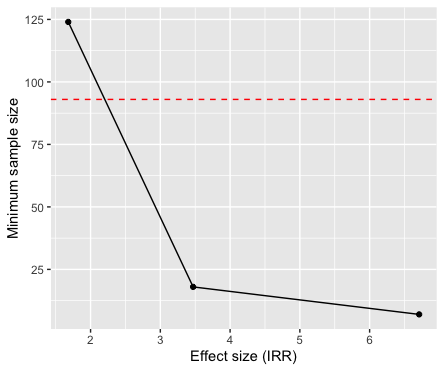
**

**REFERENCES**

1. Harms MP, Somerville LH, Ances BM, Andersson J, Barch DM, Bastiani M, et al. Extending the Human Connectome Project across ages: Imaging protocols for the Lifespan Development and Aging projects. NeuroImage. 2018;972–984.

2. Dale AM, Fischl B & Sereno MI. Cortical surface-based analysis. I. Segmentation and surface reconstruction. NeuroImage. 1999;179–194.

3. Fischl B, Sereno MI & Dale AM. Cortical surface-based analysis. II: Inflation, flattening, and a surface-based coordinate system. NeuroImage. 1999;195–207.

4. Iglesias JE, Augustinack JC, Nguyen K, Player CM, Player A, Wright M, et al. A computational atlas of the hippocampal formation using ex vivo, ultra-high resolution MRI: Application to adaptive segmentation of in vivo MRI. NeuroImage. 2015;117–137.

5. Sämann PG, Iglesias JE, Gutman B, Grotegerd D, Leenings R, Flint C, et al. FreeSurfer-based segmentation of hippocampal subfields: A review of methods and applications, with a novel quality control procedure for ENIGMA studies and other collaborative efforts. Hum. Brain Mapp. 2022;207–233.

6. Postel C, Mary A, Dayan J, Fraisse F, Vallée T, Guillery-Girard B, et al. Variations in response to trauma and hippocampal subfield changes. Neurobiol. Stress. 2021;15:15. https://doi.org/10.1016/j.ynstr.2021.100346

7. McHugo M, Talati P, Woodward ND, Armstrong K, Blackford JU & Heckers S. Regionally specific volume deficits along the hippocampal long axis in early and chronic psychosis. NeuroImage Clin. 2018;1106–1114.

8. Sahakyan L, Meller T, Evermann U, Schmitt S, Pfarr J-K, Sommer J, et al. Anterior vs posterior hippocampal subfields in an extended psychosis phenotype of multidimensional schizotypy in a nonclinical sample. Schizophr. Bull. 2021;207–218.

9. Choi S, Kim M, Park H, Kim T, Moon S-Y, Lho SK, et al. Volume deficits in hippocampal subfields in unaffected relatives of schizophrenia patients with high genetic loading but without any psychiatric symptoms. Schizophr. Res. 2022;125–131.

10. Jenkinson M & Smith S. A global optimisation method for robust affine registration of brain images. Med. Image Anal. 2001;143–156.

11. Avants BB, Epstein CL, Grossman M & Gee JC. Symmetric diffeomorphic image registration with cross-correlation: Evaluating automated labeling of elderly and neurodegenerative brain. Med. Image Anal. 2008;26–41.

12. Ashburner J & Friston KJ. Diffeomorphic registration using geodesic shooting and Gauss–Newton optimisation. NeuroImage. 2011;954–967.

13. Ridgway GR, Omar R, Ourselin S, Hill DLG, Warren JD & Fox NC. Issues with threshold masking in voxel-based morphometry of atrophied brains. NeuroImage. 2009;99–111.

14. Chen H, Cohen P & Chen S. How Big is a Big Odds Ratio? Interpreting the Magnitudes of Odds Ratios in Epidemiological Studies. Commun. Stat. - Simul. Comput. 2010;860–864.
